# Doege–Potter syndrome caused by a solitary fibrous tumor arising from the greater omentum: a case report

**DOI:** 10.1007/s12328-026-02379-0

**Published:** 2026-06-09

**Authors:** Tomoki Takizawa, Tatsuya Shonaka, Chikayoshi Tani, Hiroya Kitsunai, Hiroshi Nomoto, Mototsugu Nagao, Izumi Fukuda, Yuki Kamikokura, Mishie Tanino, Hideki Yokoo

**Affiliations:** 1 Division of Hepato-Biliary-Pancreas and Transplantation, Gastrointestinal Surgery, Department of Surgery, Asahikawa Medical University, 2-1-1-1, Midorigaoka Higashi, Asahikawa, Hokkaido 078-8510 Japan; 2 Division of Endocrinology, Metabolism, and Rheumatology Department of Internal Medicine, Asahikawa Medical University, 2-1-1-1, Midorigaoka Higashi, Asahikawa, Hokkaido 078-8510 Japan; 3 Division of Diabetology and Metabolism, Department of Internal Medicine, Tokyo Women’s Medical University School of Medicine, 8-1 Kawada-cho, Shinjuku-ku, Tokyo, 162-866 Japan; 4 Department of Endocrinology, Metabolism and Nephrology, Graduate School of Medicine, Nippon Medical School, 1-1-5, Sendagi, Bunkyo-ku, Tokyo, 113-8602 Japan; 5 Department of Diagnostic Pathology, Asahikawa Medical University Hospital, 2-1-1-1, Midorigaoka Higashi, Asahikawa, Hokkaido 078-8510 Japan

**Keywords:** Solitary fibrous tumor, Greater omentum, Doege–Potter syndrome, Non-islet cell tumor hypoglycemia, Insulin-like growth factor-II

## Abstract

Doege–Potter syndrome is a rare paraneoplastic syndrome characterized by non-islet cell tumor hypoglycemia (NICTH) caused by the excessive production of insulin-like growth factor-II (IGF-II), which occurs in less than 5% of solitary fibrous tumors (SFT). SFTs most commonly arise from the pleura, whereas primary omental origins are exceedingly rare. We report a case of Doege–Potter syndrome in an 83-year-old woman who presented with recurrent hypoglycemic episodes caused by a large primary omental SFT. Low serum insulin levels during hypoglycemia and normal adrenocortical hormone levels excluded common causes, such as insulinoma and adrenal insufficiency, and imaging findings raised the suspicion of SFT. Open surgical resection was performed, and the hypoglycemic symptoms resolved immediately postoperatively. Histopathological examination confirmed the diagnosis of high-risk SFTs. Western blot analysis of preoperative and postoperative serum samples demonstrated a postoperative reduction in tumor-derived high-molecular-weight IGF-II (big IGF-II), confirming its role as the cause of hypoglycemia. At 6 months postoperatively, no tumor or hypoglycemia recurrence was observed. This case highlights the importance of considering Doege–Potter syndrome in the differential diagnosis of intra-abdominal tumors that present with hypoglycemic episodes.

## Introduction

Solitary fibrous tumors (SFT) are rare mesenchymal neoplasms characterized by an NAB2-STAT6 fusion gene [1]. Although SFTs were originally described as tumors of pleural origin, they are now recognized to arise from virtually any anatomical site throughout the body [2]. Primary omental SFTs are exceedingly rare, with only a few cases reported in the literature [3]. Doege–Potter syndrome is a paraneoplastic syndrome associated with SFTs, in which tumor-derived high-molecular-weight insulin-like growth factor-II (big IGF-II) causes non-islet cell tumor hypoglycemia (NICTH). This syndrome occurs in less than 5% of SFT cases and is a rare clinical entity [4]. Herein, we report a case of Doege–Potter syndrome caused by a primary omental SFT. Given the extremely limited number of reported cases of hypoglycemia associated with primary omental SFT, we report this case with a review of the relevant literature.

## Case report

An 83-year-old woman presented to the emergency department with upper-extremity weakness. She was experiencing recurrent falls, anorexia, and dizziness 1 month prior to admission.She was 149 cm tall and weighed 49 kg, with a clear consciousness and stable vital signs.

On arrival her blood glucose level was 39 mg/dL, and symptoms resolved after glucose administration. Abdominal computed tomography (CT) revealed a mass approximately 20 cm in diameter extending into the pelvic cavity. The patient was admitted for further evaluation of hypoglycemia.Her medical history included hypertension and Graves’disease. She had no history of taking medications that could induce hypoglycemia, such as antidiabetic agents or corticosteroids.

During hospitalization, recurrent hypoglycemic episodes occurred but were controlled with continuous glucose infusion without requiring corticosteroid administration. Laboratory findings are presented (Table 1).Complete blood count and blood chemistry were largely within normal limits. Laboratory findings obtained during hypoglycemia (blood glucose 51 mg/dL) revealed suppressed serum insulin (IRI < 0.4 µU/mL) and C-peptide (0.19 ng/mL), thereby excluding hyperinsulinemic hypoglycemia, such as that caused by insulinoma. Plasma adrenocorticotropic hormone (ACTH) level was 32.6 pg/mL, and a rapid ACTH stimulation test demonstrated a normal response, thereby excluding adrenal insufficiency. Suppressed growth hormone (GH) and IGF-I levels raised suspicion of NICTH.

**Table 1:** Laboratory findings on admission

Test (unit)	Value
WBC (×10³/µL)	7.63
Hb (g/dL)	10.3
Plt (×10³/µL)	255
TP (g/dL)	5.6
Alb(g/dL)	2.7
T-CHO (mg/dL)	138
TG (mg/dL)	111
HDL-C (mg/dL)	34.4
LDL-C (mg/dL)	77.2
ChE (U/L)	162
AST (U/L)	25
ALT (U/L)	17
γ-GTP (U/L)	14
BUN(mg/dL)	13.1
Cre (mg/dL)	0.55
eGFR (mL/min/1.73 m²)	77.6
Na (mEq/L)	139
K (mEq/L)	4.0
Cl (mEq/L)	107
Ca (mg/dL)	8.8
Plasma glucose (mg/dL)	51
HbA1c (%)	5.2
Insulin (IRI) (µU/mL)	< 0.40
C-peptide (ng/mL)	0.19
Cortisol (µg/dL)	12.70
ACTH (pg/mL)	32.9
GH (ng/mL)	0.11
IGF-1 (ng/mL)	30
TSH (µIU/mL)	2.93
Free T3 (pg/mL)	1.97
Free T4 (ng/dL)	1.04
CEA (ng/mL)	1.7
CA19-9 (U/mL)	12
CA125 (U/mL)	83
Total ketone bodies (µmol/L)	11.2
Acetoacetate (µmol/L)	5.6
β-hydroxybutyrate (µmol/L)	5.6
Insulin antibody (U/mL)	< 0.4

**Fig. 1:**
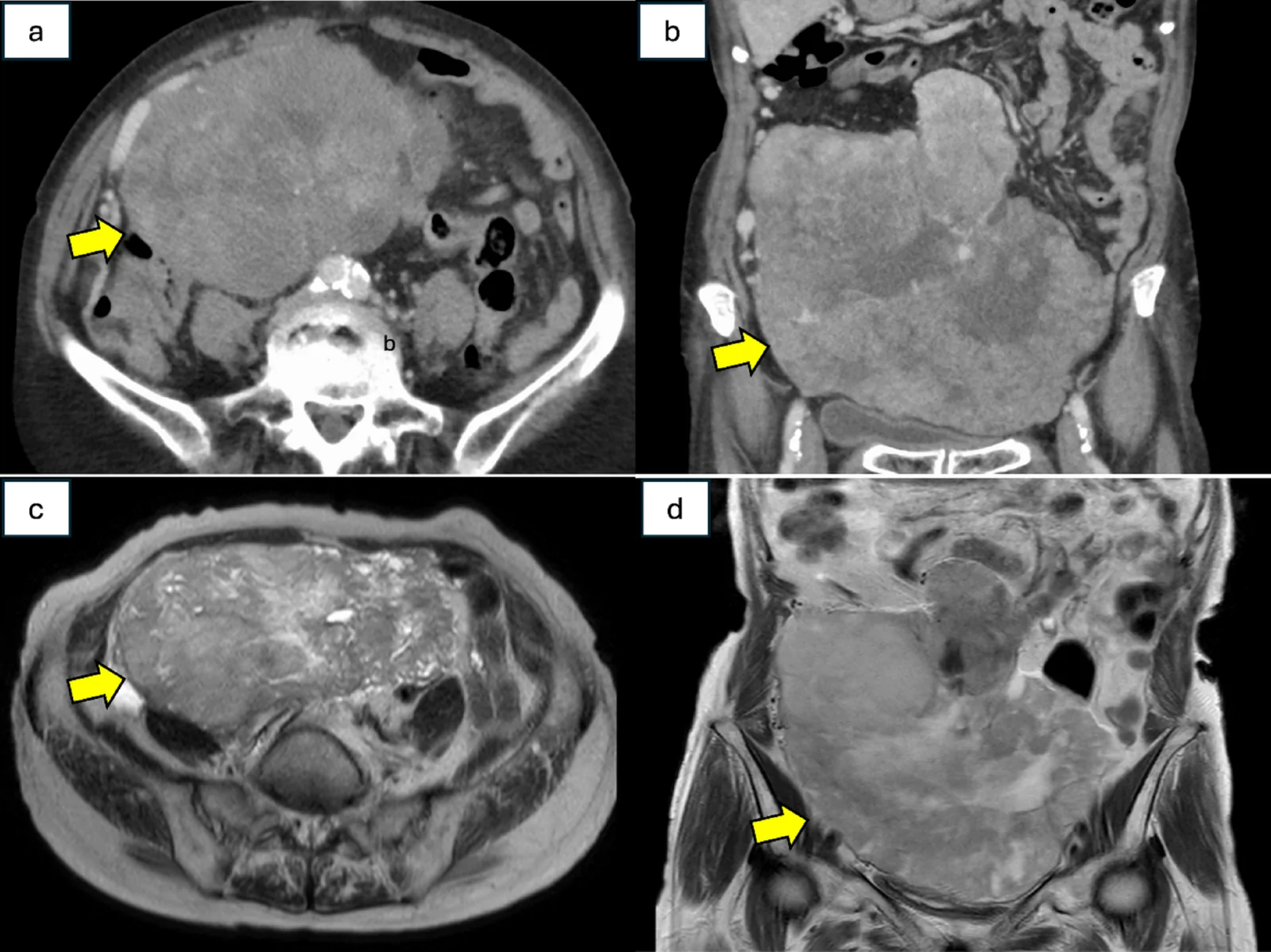
Imaging findings of the pelvic mass. Axial and coronal contrast-enhanced CT images show a large heterogeneously enhancing mass (arrows) measuring 207 × 118 × 177 mm in the pelvic cavity (**a**, **b**). Sagittal and coronal T2-weighted MRI demonstrating a solid mass (arrows) with heterogeneous signal intensity, containing mixed areas of degeneration and cystic components (**c**, **d**)

Contrast-enhanced CT revealed a mass measuring 207 × 118 × 177 mm, extending from the lower abdomen into the pelvic cavity, with a heterogeneous enhancement pattern suggesting hypervascularization (Fig. 1a, b). Magnetic resonance imaging (MRI) revealed mixed signal intensity on T2-weighted images, suggesting a solid tumor with degenerative changes, cystic components, and necrosis (Fig. 1c, d). No continuity with surrounding organs was identified. Based on these imaging findings, SFT was included in the differential diagnosis, and the patient was referred to our department for surgical management because of the suspected association between SFT and NICTH.

**Fig. 2:**
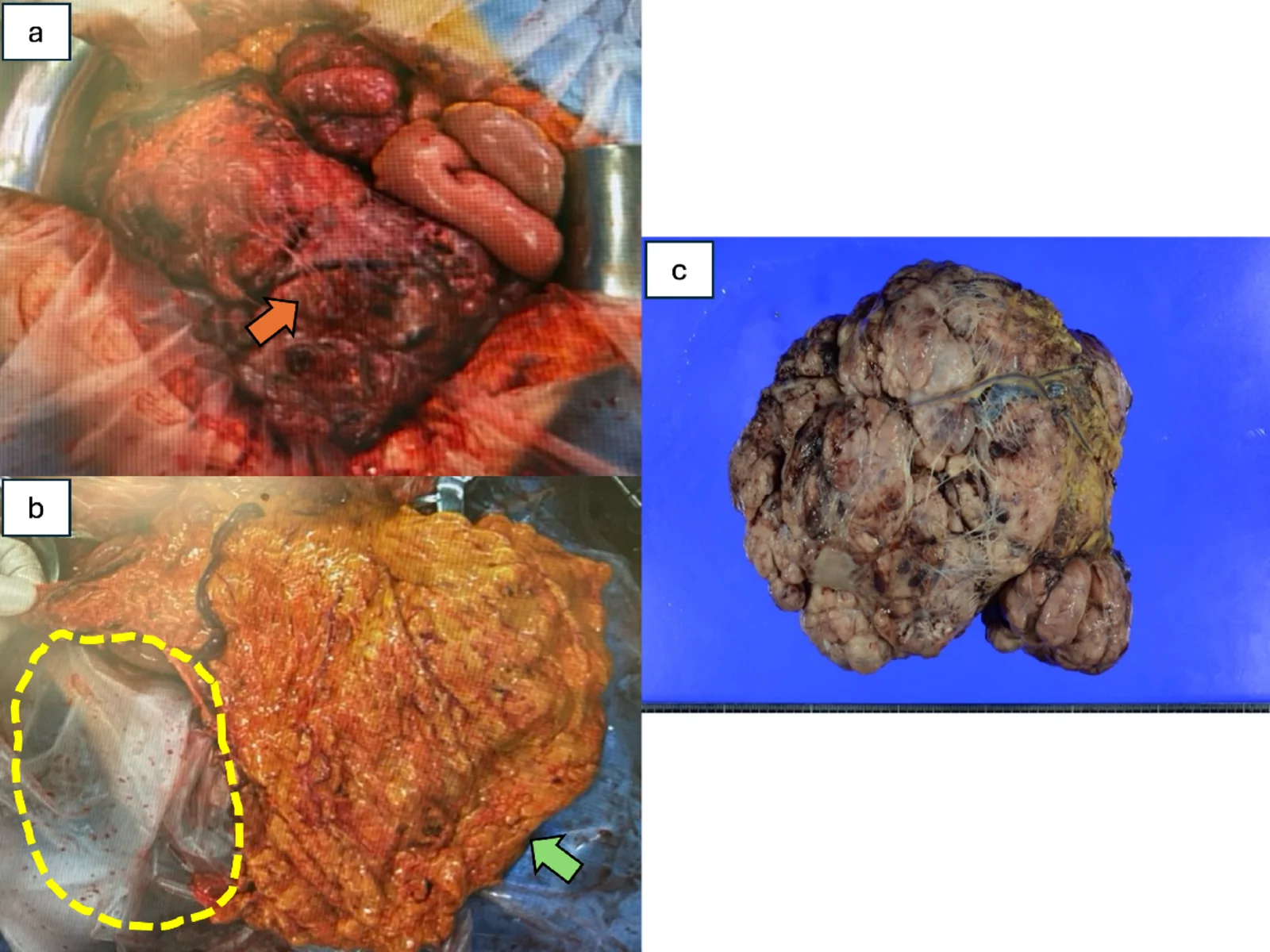
Intraoperative and gross findings of the resected tumor. Intraoperative photograph showing the tumor (orange arrow) arising from the greater omentum (green arrow), resected by local excision without adhesion to or invasion into the surrounding organs (**a**, **b**). The resection site is indicated by the yellow dashed circle. Gross specimen of the resected omental tumor measuring 210 × 200 × 140 mm (**c**)

**Fig. 3:**
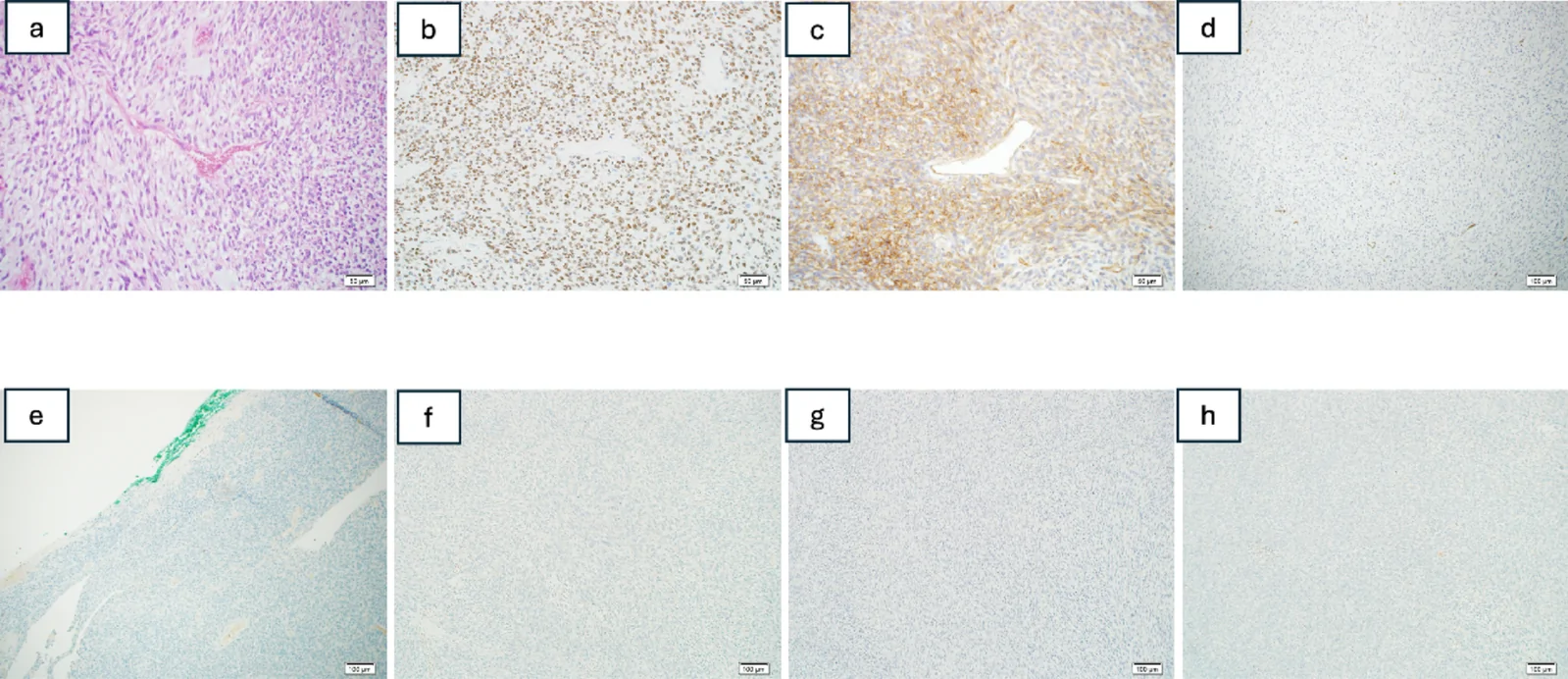
Pathological findings of the resected tumor. Hematoxylin and eosin (HE) staining (×200) showing proliferation of spindle-shaped cells arranged in fascicular and solid patterns, accompanied by scattered staghorn-shaped vessels in the stroma (**a**). The mitotic count was 20/1.734 mm², and necrosis was observed in approximately 10% of the tumor. Immunohistochemistry for STAT6 (×200) demonstrating nuclear positivity in tumor cells (**b**). Immunohistochemistry for CD34 (×200) demonstrating diffuse positivity in tumor cells (**c**). Immunohistochemistry for αSMA (×100) and S-100 protein (×100) demonstrating focal positivity (**d**, **e**). Immunohistochemistry for AE1/AE3 (×100), EMA (×100), and desmin (×100) showing negative results (**f**, **g**, **h**)

**Fig. 4:**
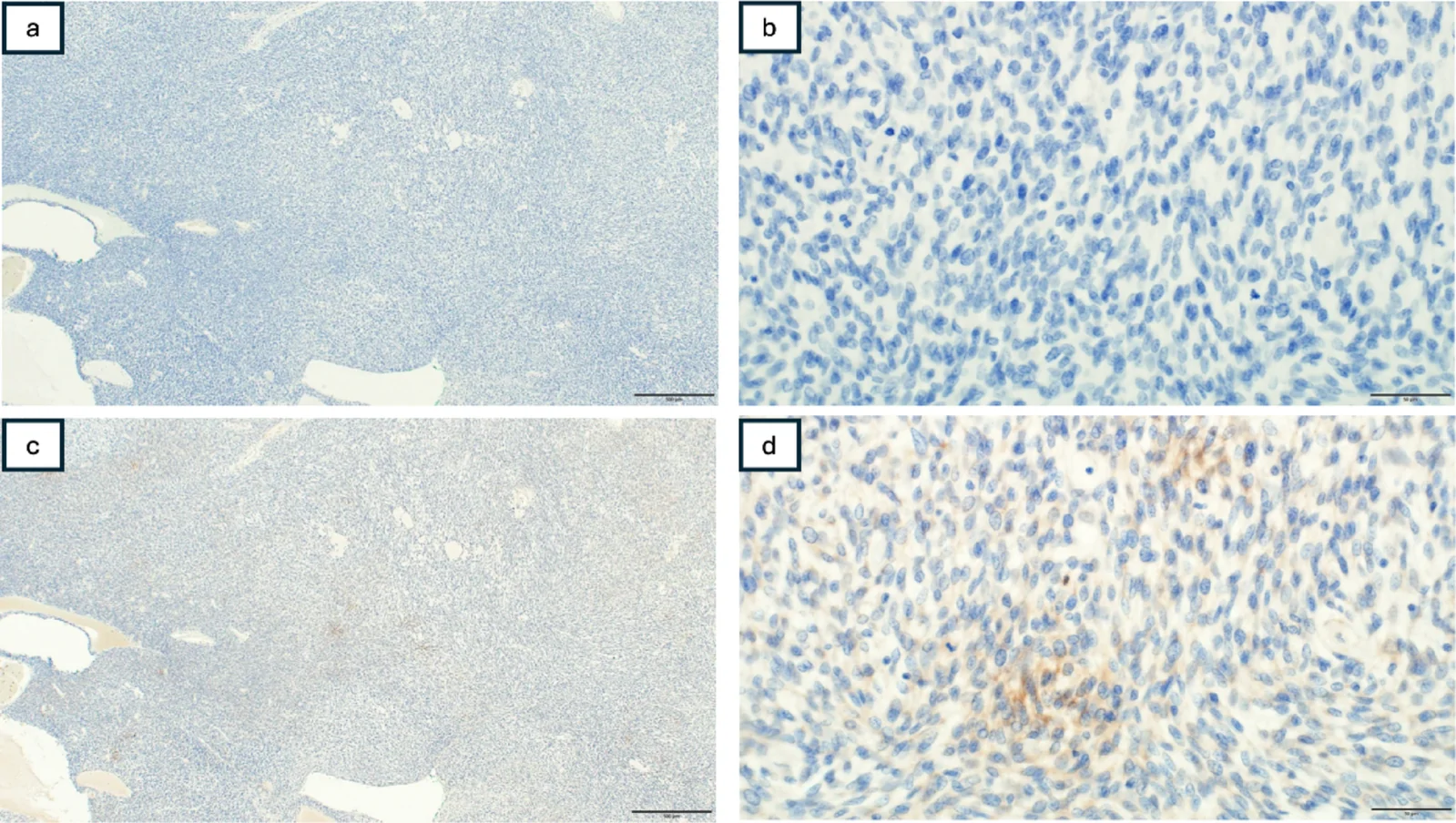
Immunohistochemistry for IGF-II of the resected tumor. Negative control showing no immunoreactivity at low (×40) (**a**) and high (×400) (**b**) magnification. Immunohistochemistry for IGF-II demonstrating focal cytoplasmic positivity in tumor cells, at low (×40) (**c**) and high (×400) (**d**) magnification, indicating IGF-II production by the tumor

Open tumor resection was performed, and the tumor was found to originate from the greater omentum without adhesion to or invasion of the surrounding organs (Fig. 2a, b). A tumor measuring 210 × 200 × 140 mm was resected (Fig. 2c). Histopathological examination showed spindle cells with relatively uniform, enlarged oval nuclei and pale eosinophilic cytoplasm proliferating in fascicular and solid patterns and covered by a fibrous capsule (Fig. 3a). Staghorn-shaped branching vessels were scattered throughout the stroma. The mitotic count was 20/1.73 mm², and necrosis was observed in approximately 10% of the tumor. Immunohistochemical staining showed nuclear positivity for STAT6 and CD34 positivity in the spindle cells, with focal positivity for αSMA and S-100, whereas AE1/AE3, EMA, and desmin were negative (Fig. 3b, c, d, e, f, g, h). Furthermore, immunohistochemical staining using an anti-IGF-II antibody revealed focal IGF-II positivity within the cytoplasm of the tumor cells (Fig. 4).

**Fig. 5:**
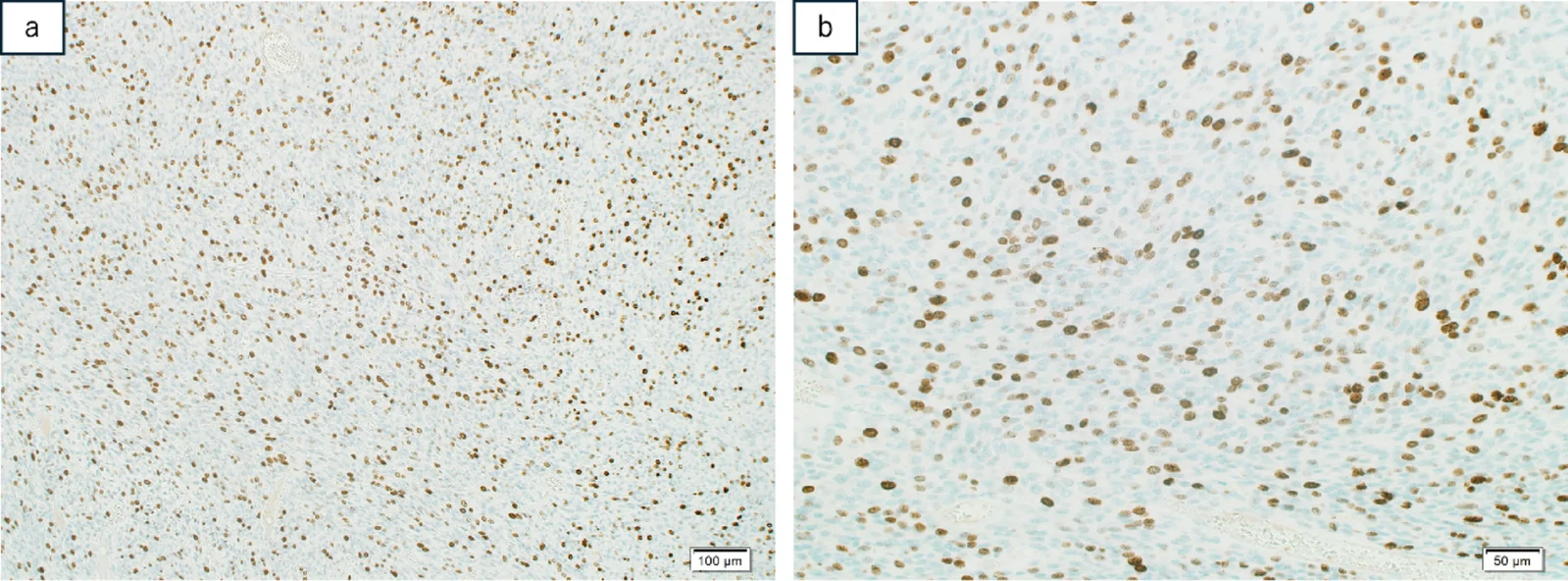
MIB-1 (Ki-67) immunohistochemistry of the resected tumor. MIB-1 (Ki-67) immunohistochemistry showing distinct nuclear positivity in tumor cells at low (×100, **a**) and high (×200, **b**) magnification. The MIB-1 labeling index was approximately 40% in the area of highest proliferation

The MIB-1 labeling index was approximately 40% in the most proliferative area (Fig. 5). These findings were consistent with a diagnosis of SFT. No vascular or lymphatic invasion was observed. Risk classification categorized the tumor as high risk.

**Fig. 6:**
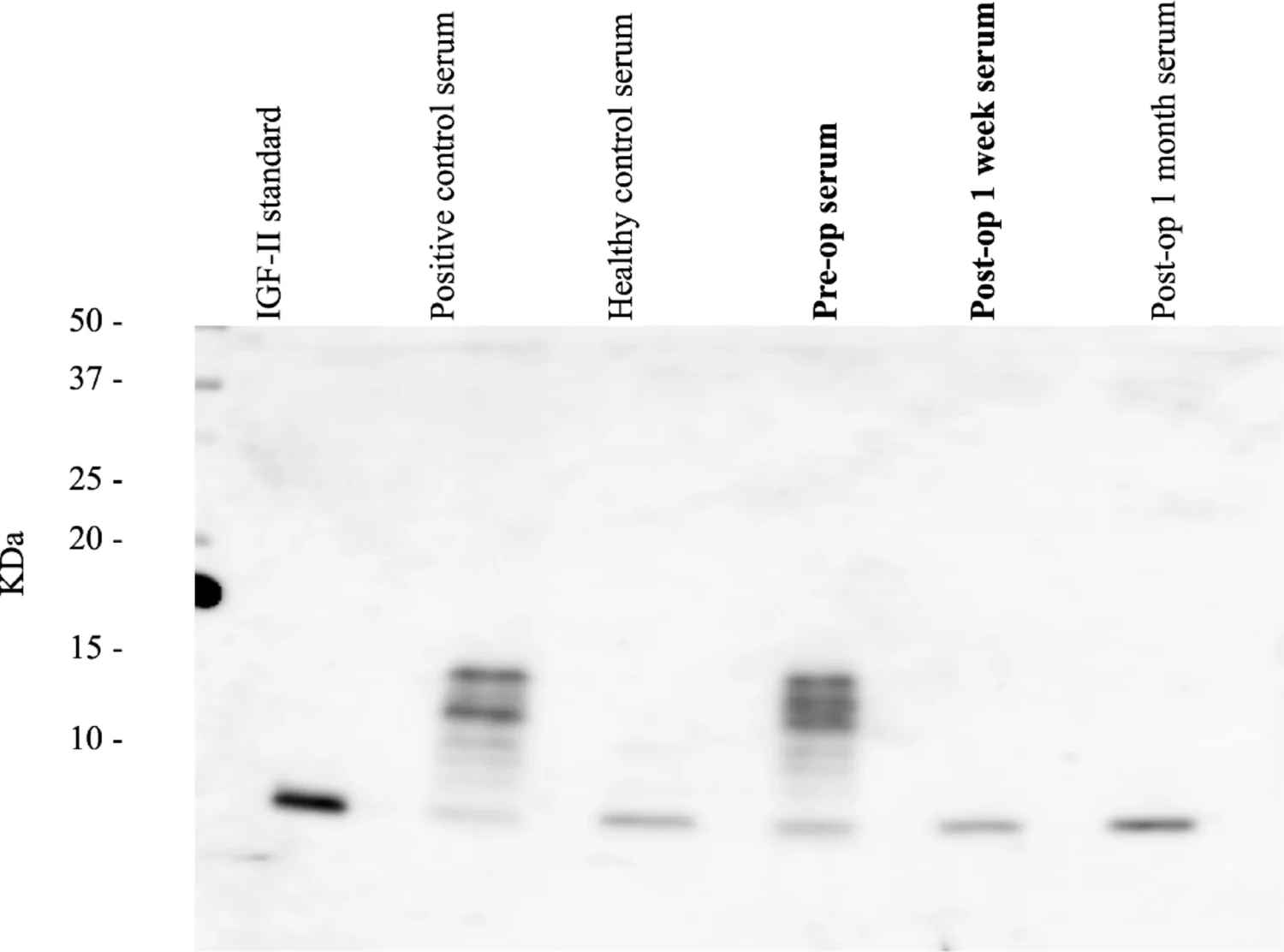
Western blot analysis for IGF-II in serum samples. Lanes from left to right: IGF-II standard; positive control serum from a patient with NICTH; control serum from a healthy subject; and, from the present case, preoperative serum, serum at one week after surgery, serum at one month postoperatively. Methods: Serum samples (equivalent to 1 µL/well) and tissue lysates (5 µg protein/well) were analyzed under non-reducing conditions. Tissue samples were homogenized in RIPA buffer, and the supernatant was processed in the same manner as the serum samples. After separation by SDS-PAGE, proteins were transferred to a membrane and blocked with 1% skim milk in TBS-T. The membrane was incubated with a primary antibody against IGF-II (clone S1F2; Merck Millipore; 1:3,000) followed by incubated with a secondary antibody (Cell Signaling Technology, #7076; 1:3,000). Signals were amplified using SuperSignal Western Blot Enhancer (Thermo Fisher Scientific, #46640) and detected with ECL Prime (GE Healthcare, #RPN2232)

Western blot analysis of preoperative and postoperative serum samples demonstrated a postoperative reduction in big IGF-II levels, with normalization of IGF-II levels (Fig. 6), confirming tumor-derived IGF-II production as the cause of the hypoglycemia.

Postoperatively, hypoglycemic episodes resolved completely. The patient had an uneventful postoperative course and was discharged on postoperative day 6. No adjuvant chemotherapy was administered, and a careful follow-up strategy was adopted. No recurrence was observed at 6 months postoperatively.

## Discussion

SFT is a rare mesenchymal neoplasm first described as a pleural tumor by Klemperer and Rabin in 1931 [1]. It has since been recognized to arise from virtually any anatomical site throughout the body, with extrapleural cases accounting for approximately 30% of all SFTs [2]. Although the abdominal cavity is among the most common sites of extrapleural SFTs, primary omental SFTs are exceedingly rare, with only a few cases reported in the literature [3].

Doege–Potter syndrome is a rare paraneoplastic syndrome in which tumors overproduce IGF-II, causing NICTH. This syndrome was independently reported by Doege and Potter in 1930 and occurs in less than 5% of SFT cases [4, 5]. In Doege–Potter syndrome, aberrant activation of IGF2 driven by EGR1 through the NAB2-STAT6 fusion gene has been reported to contribute to big IGF-II overproduction [6, 7]. Tumor-derived big IGF-II fails to form the normal ternary complex (approximately 150 kDa) and instead circulates as a smaller binary complex (approximately 50 kDa), allowing it to pass through the capillary endothelium and bind to insulin and IGF receptors, thereby exerting insulin-like effects and causing severe hypoglycemia [8, 9].

A PubMed search of Doege–Potter syndrome associated with SFTs revealed that most reported cases involved pleural lesions, followed by pelvic SFTs [5]. No cases explicitly describing a primary omental origin were identified, suggesting that the present case is exceedingly rare. Eltawil et al. reviewed 15 cases of primary omental SFT reported up to 2020, with tumor sizes ranging from 5.5 to 21 cm and a median size of approximately 10 cm [3]. Among the reported omental SFT cases, no instances of Doege–Potter syndrome or IGF-II-producing NICTH were identified. Fukuda et al. reported that tumors causing IGF-II-producing NICTH are generally large (> 10 cm) and that tumor size tends to correlate with the amount of big IGF-II produced [10]. The absence of reported Doege–Potter syndrome in primary omental SFTs may be partly attributable to the relatively small tumor sizes in previously reported cases. In contrast, the tumor in the present case measured approximately 20 cm, which may have contributed to the development of Doege–Potter syndrome.

In patients presenting with recurrent hypoglycemia, as in the present case, laboratory findings during hypoglycemic episodes are crucial for differential diagnosis. In this case, markedly suppressed C-peptide (0.19 ng/mL) and IRI (< 0.4 µU/mL) levels excluded hyperinsulinemic hypoglycemia, such as that caused by insulinoma; therefore, in cases of hypoinsulinemic hypoglycemia accompanied by a large intra-abdominal mass, NICTH should be considered in the differential diagnosis.

For the diagnosis of NICTH, confirmation of big IGF-II by Western blot analysis of serum or measurement of serum IGF-II by ELISA, with an elevated IGF-II/IGF-I ratio, is useful [11]. However, facilities capable of performing these analyses are limited; therefore, NICTH should be considered in the differential diagnosis based on clinical suspicion. In the present case, NICTH was suspected preoperatively, and Western blot analysis of preoperative and postoperative serum samples confirmed a postoperative reduction in big IGF-II levels. The combination of positive IGF-II immunostaining in the tumor tissue and complete resolution of hypoglycemic episodes after resection confirmed that tumor-derived IGF-II production was the cause of the hypoglycemia.

Histopathological examination demonstrated nuclear positivity for STAT6 and CD34 positivity, characteristic of SFTs, thereby establishing the diagnosis. STAT6 indicates the presence of the NAB2-STAT6 fusion gene and has been established as a highly sensitive and specific marker for the diagnosis of SFTs [12].

In the risk classification of SFTs, the model proposed by Demicco et al. incorporates patient age, tumor size, mitotic count, and the presence of necrosis as evaluation criteria [13, 14]. The present case exhibited multiple high-risk features, including a tumor size of 20 cm (> 10 cm), a mitotic count of 20/1.73 mm² (> 2/mm²), and necrosis (approximately 10%), resulting in a high-risk classification. High-risk SFTs carry an elevated risk of local recurrence and distant metastasis, necessitating long-term follow-up [15].

Complete surgical resection of the tumor is the definitive treatment for Doege–Potter syndrome [16]. In the present case, open tumor resection was performed, and the hypoglycemic symptoms resolved immediately postoperatively. Furthermore, the postoperative reduction in big IGF-II levels provides supporting evidence for this mechanism. In unresectable cases, medical treatments such as corticosteroid therapy, continuous glucagon infusion, and recombinant human growth hormone have been attempted; however, these approaches provide only symptomatic management [17]. Although the patient was 83 years old, the hypoglycemic episodes were life-threatening, and surgical resection was performed because her general condition was acceptable.

In this case, complete resection was achieved without macroscopic residual disease. Because the efficacy of adjuvant therapy for high-risk SFTs has not been established [18], a strategy of careful follow-up without adjuvant therapy was adopted. No recurrence was observed at 6 months postoperatively; however, because late recurrence (> 10 years) after surgery has been reported in high-risk SFT [15], long-term follow-up is essential.

In conclusion, although rare, NICTH should be included in the differential diagnosis in patients presenting with unexplained recurrent hypoglycemia, particularly those with a large intra-abdominal mass.
